# Child mental health problems as a risk factor for academic underachievement: A multi‐informant, population‐based study

**DOI:** 10.1111/acps.13426

**Published:** 2022-03-24

**Authors:** Isabel K. Schuurmans, Nathalie Tamayo Martinez, Elisabet Blok, Manon H. J. Hillegers, M. Arfan Ikram, Annemarie I. Luik, Charlotte A. M. Cecil

**Affiliations:** ^1^ Department of Epidemiology Erasmus MC University Medical Center Rotterdam Rotterdam The Netherlands; ^2^ The Generation R Study Group Erasmus MC University Medical Center Rotterdam Rotterdam The Netherlands; ^3^ Department of Child and Adolescent Psychiatry/Psychology Erasmus MC–Sophia Children”s Hospital University Medical Center Rotterdam Rotterdam The Netherlands; ^4^ Molecular Epidemiology Department of Biomedical Data Sciences Leiden University Medical Center Leiden The Netherlands

**Keywords:** child and adolescent psychiatry, epidemiology, IQ, public mental health

## Abstract

**Objective:**

To investigate whether child mental health problems prospectively associate with IQ‐achievement discrepancy (i.e., academic under‐ and over‐achievement) in emerging adolescence. The secondary aims were to test whether these associations are specific to certain mental health problems, to assess potential sex differences, and to examine whether associations are robustly observed across multiple informants (i.e., maternal and teacher‐reports).

**Methods:**

This study included 1,577 children from the population‐based birth cohort the Generation R Study. Child mental health problems at age 6 were assessed by mothers and teachers using the Child Behavior Checklist and the Teacher's Report Form. The IQ‐achievement discrepancy was quantified as the standardized residuals of academic achievement regressed on IQ, where IQ was measured with four tasks from the Wechsler Intelligence Scale for Children‐Fifth Edition around age 13 and academic attainment was measured with the Cito test, a national Dutch academic test, at the end of elementary school (12 years of age).

**Results:**

Mental health problems at age 6 were associated with IQ‐achievement discrepancy at age 12, with more problems associating with greater academic underachievement. When examining specific mental health problems, we found that attention problems was the only mental health problem to independently associate with the IQ‐achievement discrepancy (adjusted standardized difference per 1‐standard deviation, *mother*: −0.11, *p* < 0.001, 95% CI [−0.16, −0.06]; *teacher*: −0.13, *p* < 0.001, 95% CI [−0.18, −0.08]). These associations remained after adjusting for co‐occurring mental health problems. The overall pattern of associations was consistent across boys and girls and across informants.

**Conclusion:**

Mental health problems during the transition from kindergarten to elementary school associate with academic underachievement at the end of elementary school. These associations were primarily driven by attention problems, as rated by both mothers and teachers—suggesting that strategies targeting attention problems may be a particularly promising avenue for improving educational performance irrespective of IQ, although this should be established more thoroughly through further research.

**Significant outcomes**

- Poor mental health at the start of elementary school (age 6) associates with an IQ‐achievement discrepancy at the end of elementary school (age 12–13), specifically with lower academic achievement than expected based on IQ.
- Results suggest that associations between poor child mental health and academic underachievement are driven specifically by attention problems.
- Patterns of associations were generally consistent across sex and across mother and teacher‐reports, with ratings from both informants emerging as independent contributors to academic underachievement.

**Limitations**

- Poor child mental health was assessed by mothers and teachers using only partially overlapping questionnaire versions.
- The current study did not assess the bidirectional association between child mental health and the IQ‐achievement discrepancy over time, due to a lack of repeated data on academic achievement.

## 1 INTRODUCTION

The IQ‐achievement discrepancy refers to lower or higher academic achievement than expected based on IQ (i.e., academic under‐ or over‐achievement). This discrepancy is typically first observed around emerging adolescence^1^ and has been found to predict a range of individual outcomes later in life. For example, academic underachievement is associated with lower chances of academic success, increased social problems, and poorer employment prospects in adulthood.^2^, ^3^ As such, understanding which factors during the development may explain the IQ‐achievement discrepancy is essential in order to identify potential targets to maximize children's self‐actualization and chances of success.

So far, several risk factors have been identified for academic underachievement at the level of the family (e.g., early life stress),^4^ school (e.g., absence of extra‐curricular activities),^5^ and individual (e.g., poor executive functioning, psychiatric problems).^6^, ^7^, ^8^ In particular, poor mental health has been identified as an important factor associated with the IQ‐achievement discrepancy.^7^, ^9^, ^10^, ^11^ Both cross‐sectional and longitudinal studies have found that children diagnosed with disorders such as Attention‐Deficit/Hyperactivity Disorder (ADHD) and Autism Spectrum Disorder are more likely to underachieve.^7^, ^11^, ^12^ Associations have also been found in relation to subclinical mental health problems. For example, a small set of cross‐sectional studies in the general population have shown that externalizing problems (e.g.. inattention, delinquent behavior) in early childhood and late adolescence are associated with lower academic achievement than expected based on IQ.^9^, ^10^ In addition, two longitudinal studies found that early mental health problems associate with later academic underachievement and lower participation in final school examination.^13^, ^14^

While these findings suggest that child mental health is linked to academic underachievement, current research is limited in three key ways. First, population‐based studies to date have typically measured child mental health problems and IQ‐achievement discrepancy at the same time point, while only a small set of studies have examined longitudinal associations.^13^, ^14^ As such, the contribution of early child mental health to the IQ‐achievement discrepancy later in development remains largely unknown. Assessing mental health during the transition from kindergarten to elementary school may be particularly salient, as this period marks the beginning of formal education and a more structured learning environment.^15^ Indeed, adjustment to changes related to school transition has been found to play a significant role in later school success.^16^ Second, while multiple studies point to associations with attention problems, it is currently unclear to what extent these associations are independent of co‐occurring symptomatology. This is important as attention problems frequently co‐occur with internalizing and externalizing problems. Third, it is unclear whether sex moderates the association between mental health problems and the IQ‐achievement discrepancy. This is particularly relevant given the known sex differences in mental health problems, for example with boys showing more attention problems than girls.^17^, ^18^ Finally, most studies have exclusively been based on data from either parents or teachers. When assessing child behavior, a multi‐informant approach is warranted,^19^ as behavior can be variably expressed or interpreted across contexts (e.g., home versus school setting).^20^

### 1.1 Aims of the study

In the current study, we used a population‐based sample to examine the association of mental health in middle childhood (around age 6) with the IQ‐achievement discrepancy at emerging adolescence (around age 12), capturing two time points of school transition (kindergarten to elementary school, and elementary school to secondary school). We assessed (i) the association of overall child mental health, internalizing problems and externalizing problems with a later IQ‐achievement discrepancy, (ii) whether these associations are specific to certain mental health problems, (iii) whether these associations are moderated by child sex, and (iv) the consistency of associations across informants and relative importance of mother versus teacher‐reports.

## 2 METHODS

### 2.1 Study population

The current study was conducted with data from the Generation R Study, a population‐based prospective cohort from fetal life onward.^21^ In short, pregnant women were eligible for study participation if they were the residents of the study area (Rotterdam, the Netherlands), and if they had a delivery date between April, 2002, and January, 2006. A total of 9,778 mothers were enrolled. These mothers, their children and partners took part in several research waves.

A flowchart of the study population is provided in Figure S1. Of children who participated in the research center visit focused at age 13 (*N* = 4929), we excluded children who had either mother‐reported (*N* = 743) or teacher‐reported (*N* = 1824) child behavior measures not available at age 6, children who had no academic achievement measure available at age 12 (*N* = 765), or children whose IQ was not assessed at age 13 (*N* = 20). The final sample size included 1577 children.

General design, research aims, and specific measurements of the Generation R study were approved by the Medical Ethical Committee of the Erasmus Medical Center, in Rotterdam, the Netherlands, under the Declaration of Helsinki of the World Medical Association. Written informed consent was obtained from all participants. If a child was aged under 12, legal guardians gave consent on behalf of the child. When a child was aged 12 and above, written informed consent was obtained from both legal guardians and child.

### 2.2 Child mental health

Child mental health of the past 6 months was reported by both mothers and teachers. Mothers completed the Child Behavior Checklist (CBCL/1.5–5).^22^ The CBCL/1.5–5 consists of 99 items, which are scored on a three‐point Likert scale (not true, somewhat true, very true). Three overarching scales can be calculated, namely Total Problems, Internalizing Problems, and Externalizing Problems. Total Problems were calculated by summing all individual items, while Internalizing Problems and Externalizing Problems were calculated by summing individual items from a selection of subscales (i.e., *Internalizing Problems*: Anxious/Depressed, Withdrawn, Somatic Complaints, and Emotionally Reactive; *Externalizing Problems*: Aggressive Behavior and Attention Problems). In addition, seven subscales were calculated that assess specific mental health problems, being Anxious/Depressed, Withdrawn, Somatic Complaints, Aggressive Behavior, Attention Problems, Emotionally Reactive Behavior and Sleep Problems. The CBCL was filled out by primary caregivers, which was the mother in 91.2% of the cases. At the time of the CBCL, children were on average age 6.0 (standard deviation (SD) = 0.4).

Teachers completed the Teacher's Report Form (TRF/6–18).^23^ The TRF consists of 112 items, which are scored on a three‐point Likert scale (not true, somewhat true, very true). These items were also summed into three overarching scales, namely Total Problems, Internalizing Problems, and Externalizing Problems. Total Problems consists of all individual items, and Internalizing Problems and Externalizing Problems summed the individual items from a selection of subscales (*Internalizing problems*: Anxious/Depressed, Withdrawn/Depressed, and Somatic Complaints; *Externalizing Problems*: Rule Breaking Behavior and Aggressive Behavior). For the TRF, eight specific mental health problem subscales were calculated, namely Anxious/Depressed, Withdrawn/Depressed, Somatic Complaints, Aggressive Behavior, Attention Problems, Social Problems, Thought Problems, and Rule Breaking Behavior. The TRF was filled out by a total of 829 unique teachers divided over 193 unique schools. At the time of teacher‐report, children were on average of (SD = 1.4) 6.7‐year‐old.

All scales of both the CBCL and TRF were standardized and converted to T‐scores ranging from 50 to 100, using age‐ and sex‐specific norm tables. Notably, the CBCL and TRF are different, but comparable questionnaires.^24^ Therefore, a selection of scales overlap between the mother and teacher‐report (i.e., Total Problems, Internalizing Problems, Externalizing Problems, Anxious/Depressed, Withdrawn(/Depressed), Somatic Complaints, Aggressive Behavior, and Attention Problems). Both questionnaires are considered valid and reliable measures for childhood mental health problems in the clinical and non‐clinical range.^22^, ^23^

### 2.3 The IQ‐achievement discrepancy

#### 2.3.1 Intelligence quotient

Intelligence was assessed with four subtests of the Wechsler Intelligence Scale for Children‐Fifth Edition (WISC‐V).^25^ The subtests were administered by a trained examiner during the research center visit aimed at age 13 (M = 13.6, SD = 0.3). Vocabulary was administered verbally, and was used to measure verbal comprehension. Digit Span was also administered verbally, and was used to assess working memory. Matrix reasoning was administered digitally, and was used to assess fluid reasoning. Coding was administered digitally (*N* = 1534) or with a paper‐pencil version (*N* = 43), and was used to measure processing speed.

The scores on the subtests were scaled and summed, the sum score was then converted to an estimated full‐scale IQ score using a conversion table. The conversion table was created by Pearson for the Generation R Study,^26^ to provide a reliable estimate of IQ. Consistently, IQ based on this abbreviated version of the WISC‐V has a Pearson correlation of 0.93 across age 6–16 with the full‐scale IQ in other samples that also used the WISC‐V.^26^

#### 2.3.2 Academic achievement

Academic achievement of participants was assessed with the Dutch Central Institute for Test Development (Cito) test,^27^ a nationally widely used academic performance test with high reliability (Cronbach”s alpha > 0.90).^27^, ^28^ The Cito academic performance test is administered in the final year of elementary education when children are around age 12 (M = 11.8, SD = 0.4). The test consists of a total of 160 multiple‐choice questions, with which two general cognitive domains are measured. In total, 100 questions assess language skills (e.g., spelling and reading comprehension), and the remaining 60 questions assess arithmetic skills (e.g., understanding of figures and fractions). These domains are used to create a standardized score, ranging between 501 and 550. We used these standardized scores as the Cito academic performance test is adapted every school year, which makes the raw subscale scores not comparable between different years. Scores were collected by linkage through the national database (*N* = 1181) and mother‐report (*N* = 396). The two assessment types were highly correlated.^29^

#### 2.3.3 *The IQ*‐*achievement discrepancy computation*

The IQ‐achievement discrepancy is quantified using a regression approach, based on previous studies.^30^ We regressed academic achievement on IQ in a linear regression model with no additional variables. The explained variance of this regression model was high; *R* ^2^ = 0.41, *p* < 0.001. We saved the standardized residuals, which were used as a continuous measure for the IQ‐achievement discrepancy, indicating variance in academic achievement not explained by IQ. As such, the IQ‐achievement discrepancy is expressed as a z‐score, with negative values indicating how many standard deviations academic achievement is lower than expected (i.e., underachievement), and positive values indicating how many standard deviations academic achievement is higher than expected (i.e., overachievement).

### 2.4 Other variables

Sex, age during academic assessment, child ethnicity, and household income were included as covariates. Information on date of birth and sex was obtained from midwives and hospital registries. Ethnicity was acquired through maternal self‐report during pregnancy, and it was categorized according to the classification of Statistics Netherlands ^31^, which distinguishes ““Dutch”,” ““non‐Dutch Western”“ (non‐Dutch European, North‐American, and Oceanian) and “non‐Western” (Turkish, Moroccan, Indonesian, Cape Verdean, Surinamese, and Antillean). Household income was assessed using maternal self‐report at age 5 years,^21^ and has been categorized using the poverty level threshold of Statistics Netherlands into low (<1600 euro per month) and normal to high (≥1600 euro per month).^32^

### 2.5 Statistical analysis

Statistical analyses were performed with R version 3.6.3.^33^ As a first step, we performed a non‐response analysis comparing the included participants with (1) the follow‐up dataset at age 13 and (2) with the baseline sample. For our first aim, we used linear regression models to examine the association of overall mental health (i.e., Total problems, Externalizing Problems, and Internalizing Problems) with the IQ‐achievement discrepancy. We ran separate models for each scale and each informant. Our second aim focused on the association of specific mental health problems (e.g., the subscale Attention Problems) with the IQ‐achievement discrepancy. For this, we first ran separate models for each individual subscale and each informant. Then, we ran a simultaneous model that included all statistically significant subscales per informant, in order to test the independent effect of each mental health problem over and above the other mental health problems. In addition, we tested for the moderating effect of sex by rerunning models including a sex interaction with all separate mental health scales. For our last aim, we focused on scales that were reported by *both* mother and teacher (i.e., Total Problems, Internalizing Problems, Externalizing Problems, Anxious/Depressed, Withdrawn/Depressed, Somatic Complaints, Aggressive Behavior, Attention Problems). For scales that were found to be statistically significantly associated to the IQ‐achievement discrepancy in earlier analyses, we examined the unique contribution of each informant. This was done by running a mutually‐adjusted model, including the individual subscale as reported by mother, and additionally, the same subscale but then reported by teacher. As such, in these analyses, subscales reported by two informants are mutually‐adjusted.

Three post hoc analyses were executed. First, we examined whether the association between child mental health and the IQ‐achievement discrepancy was different for underachievement versus overachievement. Therefore, we categorized the IQ‐achievement discrepancy into the groups, namely: “underachieving” (≤1‐SD below the mean), “typical achieving” (within 1‐SD of the mean), and “overachieving” children (≥1‐SD above the mean). The association of the mental health scales with the achievement categories was assessed with multinomial logistic regression, with typical achieving as the reference category. Second, to increase comparability with earlier research that focused on IQ and academic achievement separately, we also assessed individual associations with IQ and academic achievement. As such, we used linear regression models to examine the association of child mental health with IQ and academic achievement as outcome. Third, as a sensitivity analyses, we assessed whether associations may have been inflated by children with an intellectual disability, for which main analyses were rerun while excluding children with an IQ lower than 75.

Beta‐coefficients from all linear regression models are interpreted as adjusted mean differences, with the beta‐coefficients representing the standardized adjusted mean difference in the outcome (i.e., expressed as z‐score) per 1‐SD increase in mental health problems. As for multiple comparisons, all *p*‐values were corrected for the effective number of tests performed. The effective number of independent tests was estimated to be 19.12 tests (ɑ = 0.003), using 100,000 random permutations that accounted for correlations between all CBCL and TRF scales. All variables had missing value frequencies below 6.2%. Missing values in covariates were imputed using Multivariate Imputation by Chained Equations (mice),^34^ using 30 imputed datasets with 60 iterations. Pooled regression coefficients were obtained using Rubin's rules.^35^

## 3 RESULTS

### 3.1 Descriptive statistics

**FIGURE 1:**
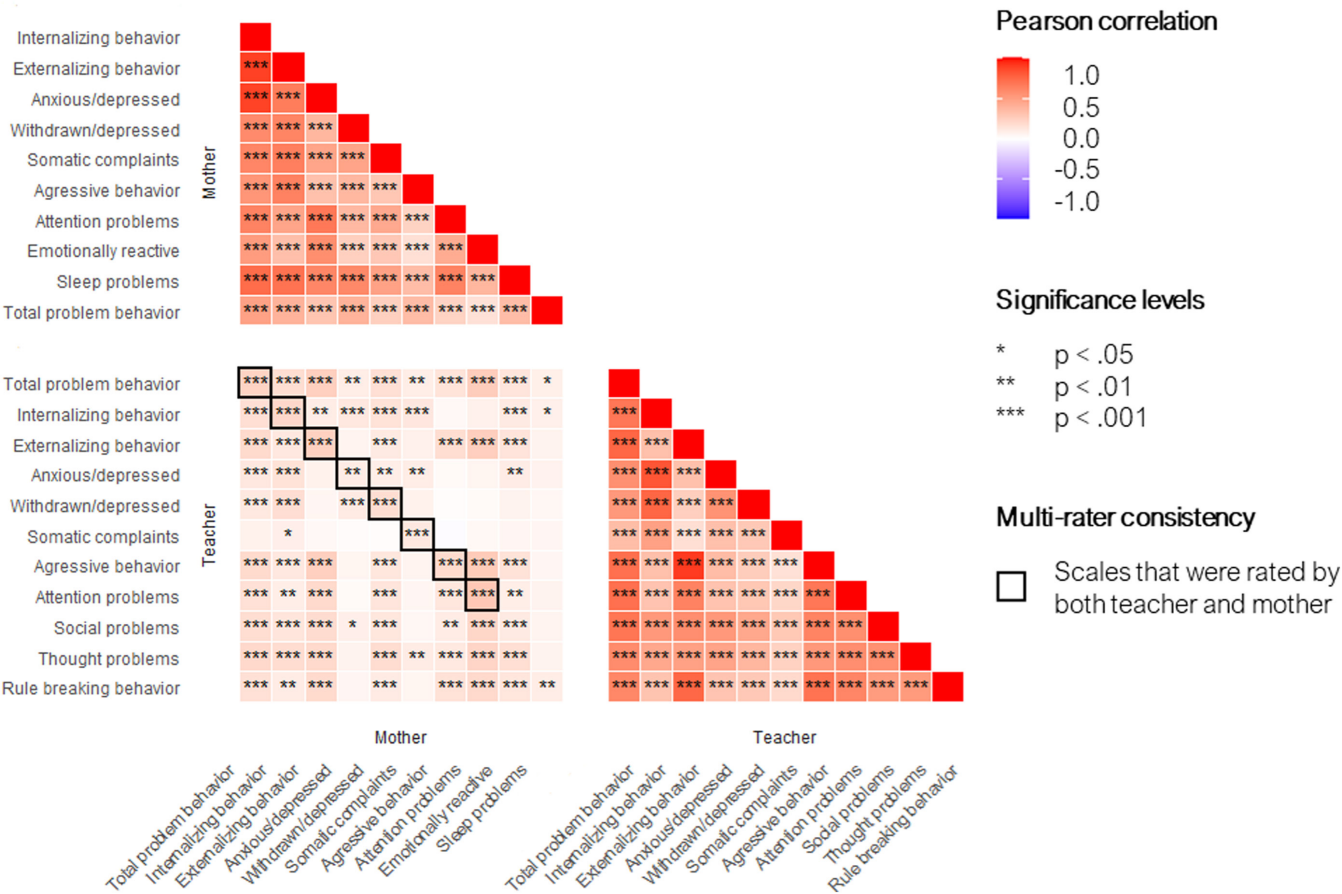
Correlation between emotional and behavioral problems

The characteristics of the study sample are shown in Table 1. A non‐response analysis can be found in Table S1. Mothers and teachers reported on a combined total of 6 overarching scales and 15 subscales, measuring a range of child mental health problems. Child mental health problems correlated more strongly within informants (Figure 1).

**TABLE 1:** Characteristics of the generation R study population

	Mean	SD	*N*	%
*Child characteristics*				
Sex				
Boy			734	47.0
Girl			843	53.0
Age child at behavioral assessment (years)				
Mother‐report	6.00	0.39		
Teacher‐report	6.74	1.37		
Age child at IQ‐achievement assessment (years)				
IQ assessment	13.57	0.29		
Academic achievement assessment	11.92	0.44		
Ethnicity				
Dutch			1059	67.2
Non‐Dutch Western			137	8.7
Non‐Western			379	24.0
Missing			2	0.1
IQ	98.95	11.68		
Academic achievement^a^	0.00	1.00		
IQ‐achievement discrepancy^a^	0.00	1.00		
*Family characteristics*				
Household income at child age 5				
Low (< 1600 euros a month)			169	10.7
Normal to high (≥ 1600 euros a month)			1399	88.7
Missing			9	0.6
Education mother at child age 5				
Low (< secondary phase 2)			140	8.9
Normal (≥ secondary phase 2 and <higher phase 2)			887	56.2
High (≥ higher phase 2)			541	34.3
Missing			9	0.6

^a^ Academic achievement and the IQ‐achievement discrepancy are standardized.

### 3.2 Overall mental health and the IQ‐achievement discrepancy

Overall, poor child mental health was statistically significantly associated with the IQ‐achievement discrepancy, with more severe symptoms being associated with greater *under*achievement relative to IQ (Table 2). Total Problems associated with the IQ‐achievement discrepancy across informants (adjusted standardized difference per 1‐SD, *mother*: −0.11, *p* < 0.001, 95% CI [−0.16, −0.06]; *teacher*: −0.14, *p* < 0.001, 95% CI [−0.19, −0.09]. Externalizing problems also associated with the IQ‐achievement discrepancy across informants (adjusted standardized difference per 1‐SD, *mother*: −0.12, *p* < 0.001, 95% CI [−0.17, −0.07]; *teacher*: −0.11, *p* < 0.001, 95% CI [−0.16, −0.06]). Substantially weaker associations were found for Internalizing Problems and the IQ‐achievement discrepancy (adjusted standardized difference per 1‐SD, *mother*: −0.08, *p* = 0.046, 95% CI [−0.12, −0.03]; *teacher*: −0.04, *p* = 1.000, 95% CI [−0.09, 0.01]).

**TABLE 2:** Association between emotional and behavior problems and the IQ‐achievement discrepancy (individual regression models)

Reporter	IQ‐achievement discrepancy			
Scale, per 1‐SD	Adjusted mean difference	95% CI	*p*	*p*‐adjusted
Mother				
Total problems	−0.11	−0.16, −0.06	<0.001	<0.001
Internalizing problems	−0.08	−0.12, −0.03	0.002	0.046
Externalizing problems	−0.12	−0.17, −0.07	<0.001	<0.001
Anxious/Depressed	−0.06	−0.11, −0.01	0.019	0.365
Withdrawn	−0.06	−0.11, −0.01	0.022	0.425
Somatic complaints	−0.08	−0.13, −0.03	0.002	0.042
Aggressive behavior	−0.06	−0.11, −0.01	0.019	0.368
Attention problems	−0.11	−0.16, −0.06	<0.001	<0.001
Emotionally reactive behavior	−0.05	−0.10, −0.01	0.028	0.544
Sleep problems	−0.07	−0.12, −0.02	0.005	0.098
Teacher				
Total problems	−0.14	−0.19, −0.09	<0.001	<0.001
Internalizing problems	−0.04	−0.09, 0.01	0.106	1.000
Externalizing problems	−0.11	−0.16, −0.06	<0.001	<0.001
Anxious/Depressed	−0.04	−0.09, 0.01	0.125	1.000
Withdrawn/Depressed	−0.01	−0.06, 0.04	0.620	1.000
Somatic complaints	−0.04	−0.09, 0.01	0.137	1.000
Aggressive behavior	−0.10	−0.15, −0.05	<0.001	<0.001
Attention problems	−0.13	−0.18, −0.08	<0.001	<0.001
Social problems	−0.06	−0.11, −0.01	0.011	0.218
Thought problems	−0.07	−0.12, −0.02	0.007	0.128
Rule breaking behavior	−0.09	−0.14, −0.04	<0.001	<0.001

Note

Adjusted standardized mean differences represent beta‐coefficients from linear regression models, expressing a standardized difference in the IQ‐achievement discrepancy per 1‐SD more mental health problems. Each row corresponds to the output of the independent contribution of parent‐ and teacher‐reported subscales, separately. Negative estimates indicate that higher problem behavior is associated to lower actual achievement than would be expected based on IQ. Estimates are standardized. All models adjusted for age at academic assessment, sex of the child, and household income.

### 3.3 Specific mental health problems and the IQ‐achievement discrepancy

When examining specific mental health problems, we found that associations for mother‐report were statistically significant for Attention Problems and for Somatic Complaints (see Table 2), after multiple testing correction. For teacher‐reported subscales, we found statistically significant associations for Attention Problems, Aggressive Behavior, and Rule Breaking Behavior (see Table 2), after multiple testing correction.

In a simultaneous model that included the two statistically significant mother‐reported subscales, only Attention Problems remained statistically significantly associated with the IQ‐achievement discrepancy (adjusted standardized difference per 1‐SD, *mother*: −0.10, *p* = 0.003, 95% CI [−0.15, −0.05]). Similarly, in a simultaneous model that included all statistically significant teacher‐reported subscales, Attention Problems was the only subscale that remained statistically significantly associated (adjusted standardized difference per 1‐SD, *teacher*: −0.12, *p* = 0.015, 95% CI [−0.19, −0.05]). When testing for the moderating effect of sex (Figure S2), only Thought Problems showed a nominally statistically significant sex moderation, but this finding did not survive multiple testing correction.

### 3.4 The unique contribution of reporters

In a mutually‐adjusted model that included both mother‐reported and teacher‐reported Total Problems in one model, mother‐report and teacher‐report both were independently statistically significantly associated with the IQ‐achievement discrepancy (adjusted standardized difference per 1‐SD, *mother*: −0.09, *p* = 0.007, 95% CI [−0.15, −0.04]; *teacher*: −0.13, *p* < 0.001, 95% CI [−0.19, −0.09]). Similarly, mother‐reported and teacher‐reported Externalizing Problems both were statistically significant associated to the IQ‐achievement discrepancy in a mutually‐adjusted model (adjusted standardized difference per 1‐SD, *mother*: −0.11, *p* = 0.001, 95% CI [−0.16, −0.06]; *teacher*: −0.09, *p* = 0.007, 95% CI [−0.15, −0.04]). Finally, in the model that included mother‐rated and teacher‐rated Attention Problems, both remained independently statistically significant associated to the IQ‐achievement discrepancy (adjusted standardized difference per 1‐SD, *mother*: −0.09, *p* = 0.024, 95% CI [−0.14, −0.03]; *teacher*: −0.11, *p* < 0.001, 95% CI [−0.17, −0.06]).

### 3.5 Post hoc analyses

**FIGURE 2:**
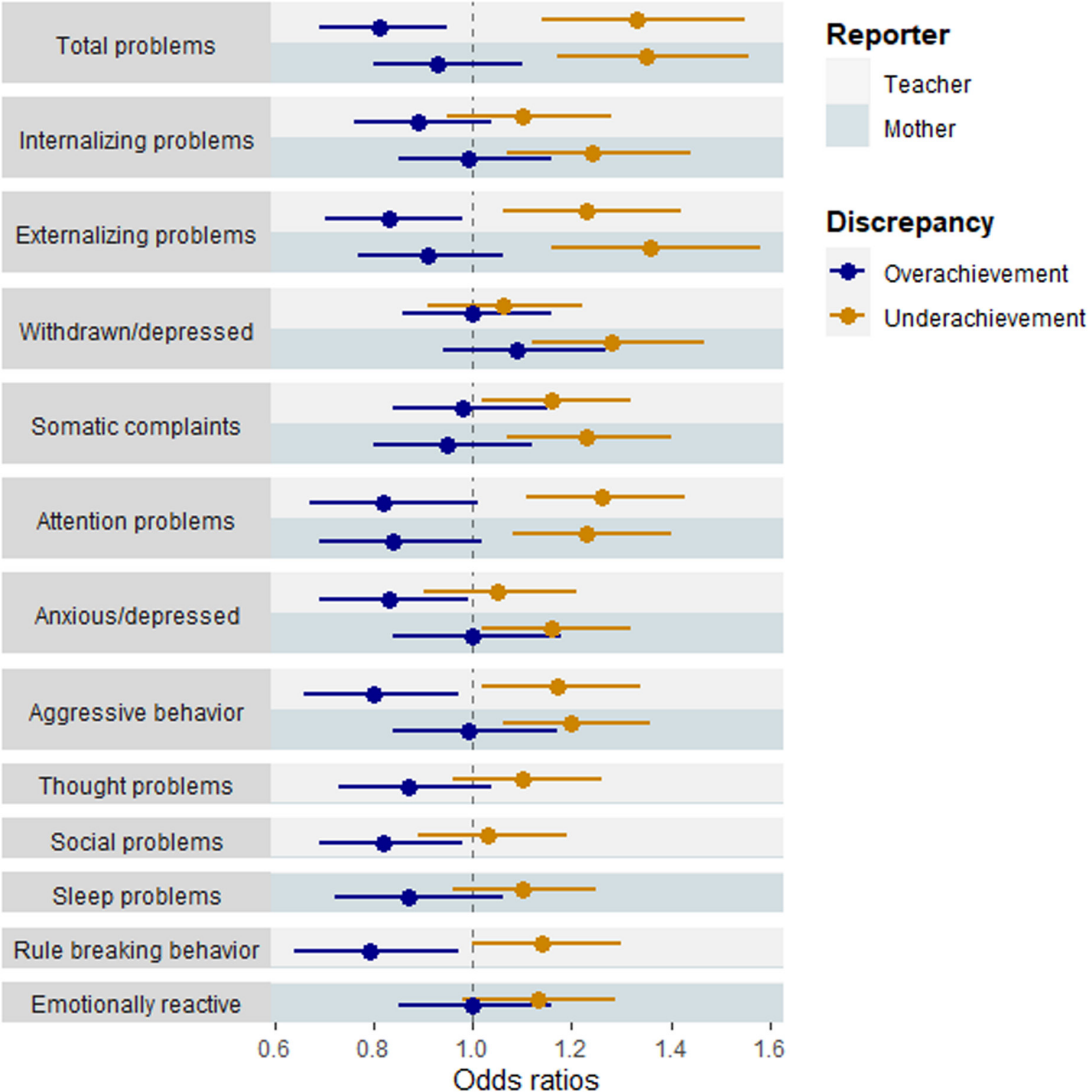
Association between emotional and behavior problems and underachievement or overachievement Each row corresponds to the output of the independent contribution of parent‐ and teacher‐reported subscales, separately. Underachievement is defined as ≤1‐SD lower academic achievement than expected, overachievement is defined is ≥1‐SD higher academic achievement than expected. Odds ratios reflect the comparison of the odds of typical achievement to the odds of overachievement *or* underachievement

Categorical analyses were performed to examine the association of mental health problems with typical achievement versus both underachievement and overachievement. Consistent with a linear association between mental health and achievement, we found that compared with typical achievement, increased poor child mental health was associated with higher odds for *under*achievement and lower odds for *over*achievement (Figure 2).

Analyses were repeated with IQ and academic achievement as separate outcomes (Table S2). The majority of child mental health problems reported by either mothers or teachers were statistically significantly associated with lower academic achievement. In contrast, only mother‐ and teacher‐reported Attention Problems, mother‐reported Externalizing Problems, and teacher‐reported Total Problems statistically significantly associated with lower IQ.

Finally, as a sensitivity analyses models were rerun while excluding children with an IQ lower than 75. Results were largely consistent with the initial analyses (Table S3).

## 4 DISCUSSION

In this population‐based study, we investigated how mental health in middle childhood (around age 6 years) associates with the IQ‐achievement discrepancy in emerging adolescence (around age 12 years)—two time periods characterized by crucial school transitions (i.e., kindergarten to elementary school, and elementary school to secondary school). We highlighted three key findings: first, overall mental health problems associated with later academic underachievement. Second, these associations were specifically driven by attention problems, which was found to associate with the IQ‐achievement discrepancy above and beyond other co‐occurring mental health problems. Third, patterns of associations were generally consistent across boys and girls and also across mother and teacher‐reports, with both informants emerging as independent contributors to the IQ‐achievement discrepancy.

Our findings suggest that children with poor overall mental health at the beginning of elementary school are more likely to show an IQ‐achievement discrepancy at the end of elementary school, specifically academic underachievement. The associations were found for total problem behavior and externalizing problem behavior, but weak evidence was found internalizing problems with no evidence for an effect in teacher‐report in the association with the IQ‐achievement discrepancy. In addition, overall mental health was found to associate with lower academic achievement and to a lesser extent lower IQ separately over time. These findings are in line with previous literature that consistently found child mental health to be cross‐sectionally associated (i) with lower academic achievement than expected based on IQ,^9^, ^13^ (ii) with lower IQ,^36^ and (iii) with lower academic achievement.^14^, ^37^, ^38^ Our study extends these findings by identifying *long*‐*term effects* of poor mental health on academic underachievement in the general population. Middle childhood coincides with a time when important foundations for school performance are laid.^39^, ^40^ Mental health problems at this age may interfere with the acquisition of academic skills, leading to difficulties that persist during elementary school. Future research with repeated measures is needed to clarify how the association between child mental health and the IQ‐achievement discrepancy unfolds over time, and whether associations are bidirectional. This is warranted, given that academic underachievement may also exacerbate mental health problems, for instance, because increased stress, lower self‐esteem, and decreased feelings of usefulness accompanied with completing tasks.^2^

When investigating specific mental health problems, we found that observed associations were driven specifically by attention problems, consistent with previous literature.^7^, ^9^, ^10^, ^11^, ^12^ We build on this evidence by showing that this association is independent of co‐occurring mental health problems, is observed over a time period of 6 years in a large multi‐ethnic cohort and is consistent across multiple informants. Further, despite sex differences in attention problems and academic underachievement,^17^, ^18^ we found comparable associations for boys and girls. Of note, we used a sex‐normed questionnaire to assess mental health, which inherently reduces sex differences. In addition, the distribution of subclinical mental health problems shows fewer sex differences than does the sex distribution on clinical diagnoses (i.e., ADHD).^18^ In addition, large power is typically needed for identifying sex moderation. We for instance found a nominally statistically significant sex moderation with thought problems, but this finding did not survive multiple testing correction, indicating that power might have been insufficient in our sample. Nonetheless, both boys and girls with attention problems may particularly benefit from early childhood interventions that enable them to perform in accordance with their capacity, for instance from psychosocial interventions that improve educational functioning by targeting both family and school.^41^ There are several explanations why specifically attention problems may affect the IQ‐achievement discrepancy as opposed to other problem behaviors. For example, the association could be mediated through executive functioning.^42^ Low executive functioning has been implicated in attention problems, and also has been suggested to be particularly important in the IQ‐achievement discrepancy.^6^, ^8^ Moreover, teachers report that they feel less emotionally connected with children with ADHD, a mental disorder characterized by attention problems, and also report that students with ADHD are more challenging to work with.^43^ This low teacher‐student bond could be harmful, as reduced teacher acceptance is associated to lowered academic achievement.^44^ An alternative explanation of the association between attention problems and academic underachievement could be that academic tasks require sustained allocation of attention, which may be particularly difficult for children with attention problems.^45^ Accordingly, children with ADHD may not have problems with classroom content, but rather with classroom processes such as school motivation and study skills.^46^ This in turn could affect school achievement.^47^ However, cognitive difficulties in children diagnosed with ADHD may not reflect those of children with attention problems in the general population. Nonetheless, the finding that children with attention problems are at increased risk underachievement is of concern. Firstly, because attention problems are highly common, affecting approximately 2–3% of school‐going children^48^, ^49^; and secondly, because academic underachievement itself is associated with a range of negative outcomes later in life, such as poorer employment prospects, health and higher mortality.^50^

Mother and teacher‐reports showed comparable associations between child mental health and academic underachievement. This is noteworthy given that informant reports are typically only modestly correlated, as reporters might differ on how they perceive behavior.^51^ Nonetheless, when parent–teacher concordance in child behavioral ratings are high, the outcomes are generally found to be more severe.^52^ Of note, our findings for mother‐report differ from those reported by previous studies,^9^ who only identified statistically significant associations of teacher‐report with the IQ‐achievement discrepancy. However, their study assessed only cross‐sectional association between problem behavior and the IQ‐achievement discrepancy at age 6 years.^9^ A possible explanation for the contradictory result could be that maternal reports are not associated with the IQ‐achievement discrepancy directly at an early age, which may also be also harder to detect during this period as the discrepancy is typically first observed around emerging adolescence.^1^ Moreover, inconsistencies in results could reflect differences in participant characteristics (e.g., rural/ primarily Caucasian sample vs. urban/ ethnically mixed sample). Further, our sample size is considerably larger, which gives us more power to detect small effects. Overall, the association between child mental health symptoms and the IQ‐achievement discrepancy is observed across different settings/reporters, adding confidence to the robustness of our associations.

The present findings should be interpreted in light of a number of limitations. First, a substantial amount of our original sample was excluded due to missing data. Participation was higher in participants with a more favorable socioeconomic status, lifestyle, and health. This may have led to selection bias, particularly as the prospective nature of the study increases the risk of loss to follow‐up.^53^ Second, different questionnaire versions were used for the CBCL and TRF, respectively, the preschool version (aimed at 1.5–5 years) and the school‐age version (aimed at 6–18 years). The Internalizing and Externalizing Problems scales used across informants include different subscales for each version, as for instance the Externalizing Problems scale only includes the Attention Problems for the mother‐report (CBCL), but not for the teacher‐report (TRF). Nonetheless, the different versions measure overlapping scales, and standardized T‐scores were used to make these scales more comparable. Third, a shortened version of the WISC‐V was used; however, the association between the shortened and the full version of the WISC‐V is high.^26^ Fourth, the IQ‐achievement discrepancy is calculated by predicting academic achievement at age 11.9 with IQ measured at age 13.6, meaning the discrepancy is predicted backward in time. Although IQ is known to be stable over time,^54^ it would have been more optimal to have both measures at the same time point. Fifth, due to the lack of data on the IQ‐achievement discrepancy at an early age, we were only able to examine the association from early child behavior to a later IQ‐achievement discrepancy. It would be of interest to also assess the association from an early IQ‐achievement discrepancy to later mental health problems, particularly as previous work found early IQ‐achievement discrepancies to be associated with poorer mental well‐being, suggesting bidirectional associations.^2^, ^55^ As for our strengths, we used multiple informants for the child behavior assessment, improving the validity and robustness of our findings. In addition, we had a large population‐based sample, enabling inferences from our results to the general population. Moreover, we used objective and standardized tests to estimate the IQ‐achievement discrepancy. Namely, in the Netherlands, children can enter one of the four different types of secondary education (i.e., low, intermediate, high preparatory vocational, and pre‐university), which represent different levels of academic difficulty.^56^ We used an academic achievement test that is widely used in Dutch elementary schools to make an official recommendation regarding the secondary school level that is most appropriate to students their academic abilities.^27^ More details on the Dutch educational system can be found elsewhere.^57^

In conclusion, in this population‐based study, we identified poor child mental health as a potential developmental risk factor for later IQ‐achievement discrepancy, using a Dutch real‐life academic achievement measure that plays a decisive role in children's academic trajectory. When examining specific mental health problems, we found that attention problems were independently associated with lower academic achievement than would be expected based on IQ, over and above other mental health problems. These associations were robust across multiple informants, and comparable for boys and girls. Together, these findings point to attention problems during the transition from kindergarten to elementary school as an important early risk factor for academic underachievement in the general population, and a potential important target for helping children meet their full academic potential.

## CONFLICT OF INTEREST

All authors have declared having no competing or potential conflicts of interest.

## AUTHOR CONTRIBUTIONS

IS, AI, and CC designed the study. IS, NTM, and CC had full access to the data and were responsible for data integrity and statistical analyses. IS, AI, CC, NTM, and EB interpreted the data. IS wrote the manuscript and integrated feedback from other authors. All authors were involved in critical revision of the study design and manuscript drafts and approved the final version.

### PEER REVIEW

The peer review history for this article is available at https://publons.com/publon/10.1111/acps.13426.

## Supporting information

Supinfo

## Data Availability

The data used in this study can be acquired upon request. Requests should be directed to the management team of the Generation R Study ( secretariaat.genr@erasmusmc.nl ), which has a protocol of approving data requests. Because of restrictions based on privacy regulations and informed consent of participants, data cannot be made freely available in a public repository.
